# Shannon D. Jones, MLS, MEd, AHIP, FMLA, Medical Library Association President, 2022-2023

**DOI:** 10.5195/jmla.2022.1634

**Published:** 2022-10-01

**Authors:** Kelsa Bartley, Tamara M. Nelson, Jamia Williams, Aidy Weeks

**Affiliations:** 1 k.bartley@med.miami.edu, Assistant Professor, Outreach and Education Librarian, Louis Calder Memorial Library, University of Miami Miller School of Medicine; 2 tnelso24@uthsc.edu, Associate Professor, User Services Coordinator/Senior Research & Learning Services Librarian, University of Tennessee Health Science Center; 3 jamia.williams@utah.edu, Consumer Health Program Specialist, The Network of the National Library of Medicine Training Office, University of Utah, Salt Lake City, Utah; 4 aidybert.weeks@unlv.edu, Assistant Professor, Director, School of Medicine Library, University of Nevada, Las Vegas, Las Vegas, NV.

## Abstract

In this profile, Shannon D. Jones, MLS, MEd, AHIP, FMLA, Medical Library Association President, 2022-2023, MJ Tooey describes her as someone who “takes chances on people, valuing those others might not see as valuable”. Jones embraces lifelong learning, and it shows up in her collegiate journey; she has been a student of leadership, a leader of institutions, especially within the Medical Library Association (MLA); and a leader in librarianship. She is a trailblazer, the second African American MLA president, and a champion of diversity, equity, inclusion, and belonging. Jones has been Director of Libraries & Professor at the Medical University of South Carolina (MUSC) for the past seven years and is also Director of Region 2 of the National Network of Libraries of Medicine, National Library of Medicine.

**Figure:**
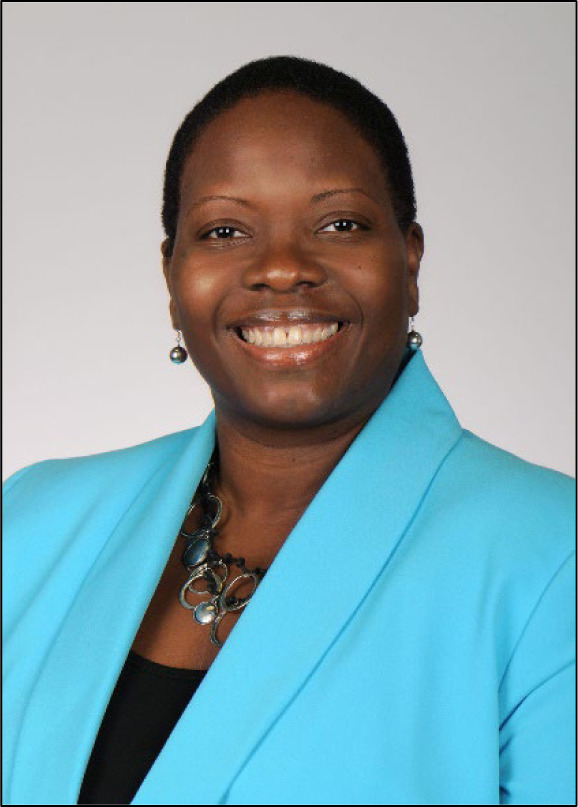


## INTRODUCTION

Shannon D. Jones, AHIP, FMLA, began her career in health sciences as an Associate Fellow for the United States National Library of Medicine (NLM) in 2002 and later went on to accept a position at Tompkins-McCaw Library for Health Sciences (now the Health Sciences Library), Virginia Commonwealth University Libraries in Richmond, VA which was her host site for her second year as an NLM Fellow. During her tenure at VCU (2004-2014), she served as Education Services Outreach Librarian, Head of Outreach Services, and Associate Director, Research and Education. In 2014, Shannon joined the Medical University of South Carolina faculty in the role of Assistant Director for Program Development and Resource Integration before being promoted to her current role as Director of Libraries in 2015. In 2021, she was also named Director of Region 2 for the National Network of Libraries of Medicine, National Library of Medicine (NNLM). Shannon is the first African American to hold the NNLM regional medical library director title.

Shannon is a native of Norfolk, VA, and as a first-generation college student, she received her Bachelor of Arts in English from North Carolina State University. She went on to receive both a Master's in of Library Science and Master's in of Information Science from North Carolina Central University, as well as a Masters of Education in Adult Learning with an emphasis in Human Resource Development from Virginia Commonwealth University. Shannon is currently nearing completion of an Education Doctorate in Educational Leadership from Charleston Southern University.

Shannon credits her grandmother, who was born on a farm and had a seventh-grade education, for her love of libraries and learning. Her grandmother was born during a period when African Americans were not allowed to pursue an education, and their career options were limited. Therefore, she strongly pushed for her grandchildren to get an education and do well in life. Her mother also influenced her decision to become a librarian. She taught her to be confident in her ability to do anything she wanted to do and pursue her dreams regardless of what others may say. Instilled with her grandmother and mother's wisdom, traditions, and legacies, Shannon ferociously set out to become the best librarian she could be.

She found her passion for medical librarianship after landing a position at Eastern Virginia Medical School. She was enthralled by the way librarians interacted with clinical teams and how it impacted patient care. Shannon quickly realized the impact of medical librarianship on educating future healthcare professionals, aiding in their clinical decision making. This was just the beginning of what would become a stellar career in medical librarianship.

## A STUDENT OF LEADERSHIP

### NLM Association Fellow

After graduating in 2002 with her Master of Library Science from North Carolina Central University in Durham, North Carolina – part of the academic mosaic of established Historically Black College & Universities (HBCUs) – Shannon set her sights on applying to the Associate Fellowship Program at the National Library of Medicine (NLM) in Bethesda, Maryland. Established as a year-long internship for newly graduated librarians in 1957, the Associate Fellowship program offers a residency and field placement to postgraduates interested in entering medical and health sciences librarianship [1]. Shannon would apply and later be accepted to the 2002-2003 NLM Associate Fellow cohort and pursue a variety of professional interests, including “community and disease-specific outreach programs, medical informatics, emerging technologies in libraries, consumer health, web-based outreach services, and the dissemination of information to underserved populations” [2].

Shannon not only brought her information sciences knowledge to the Fellows program but also her lived experience as a Black, Indigenous, Person of Color (BIPOC) woman having first-hand knowledge as a caregiver to family members who had experienced systemic healthcare barriers, including her mother who had endured and sadly lost the battle to diabetes during Shannon's time in the program. With her tenacity to be action-oriented, a curious learner,and an advocate for engaging historically excluded groups, Shannon expanded her health sciences information knowledge at the NLM. During her tenure as a Fellow, Shannon completed a bibliography project on forensic science for K-12 students, a data mining project within the Public Services Division, a usability design project to improve the Electronic Fund Transfer System known as EFTS, and provided community outreach to African American teens through the Youth Healthbuilders Corps and the NNLM National Network Office and Office of Health Information Programs Development [3][4].

Shannon would later remark that participating in the Fellows Program allowed her to see the “…essential role that the National Library of Medicine play[ed] in supporting librarians and libraries of all types…” for Shannon, being an Associate Fellow “…exposed me to the people, resources, information products, and services that became mainstays when I assumed my first position as an instruction librarian” [5]. The Associate Fellows program made it clear to Shannon the importance of medical and health sciences librarians as vital to the research, instruction, and engagement pillars that are foundational to the success of any academic health sciences library and the communities it supports.

### NLM/AAHSL Leadership Fellow

After concluding the Fellows program in 2004, Shannon began her first academic health sciences librarian role as the Education Services Outreach Librarian for the Tompkins McCaw Library (TML) for the Health Sciences at Virginia Commonwealth University (VCU). Within six years, Shannon rose through the leadership ranks of VCU, and by 2010, Shannon had served as interim head of Education Services and, finally, the Associate Director of Research and Education. In this role, Shannon managed library operations both in the Community Health Education Center and the TML Learning Center at Hunton, evaluated and assessed research and outreach programming, and led a team of 8 faculty librarians, 4 classified staff, and a team of student workers. In looking for opportunities to continue her growth as an academic leader, Shannon applied and was accepted to the 2011-2012 cohort of NLM/Association of Academic Health Sciences Libraries (AAHSL) Leadership Fellows program.

Similar to the NLM Associate Fellows Program, the NLM/AAHSL Leadership Fellows is open to librarians in the medical and health sciences library field who are interested in becoming library directors and are paired with existing library directors with the opportunity to visit their home institution [6]. During her tenure as a Leadership Fellow, Shannon was mentored by Regina “Kenny” Marone, who at the time was the Director of the Cushing/Whitney Medical Library and Associate University Librarian for School and Department Libraries at Yale University [7].

### Other Professional Development Opportunities in Leadership

Shannon's leadership journey also included opportunities to participate in several other professional development programs, including the Harvard Leadership Institute for Academic Librarians and the Association of Research Libraries (ARL) Leadership and Career Development Program (LCDP) [8]. As an MLA scholarship recipient, Shannon was part of the ARL LCDP Renaissance Class for 2007-2008, conducting a research project that evaluated outreach efforts in digitally-focused academic health sciences libraries with mentor William “Bill” Walker, who at the time was Dean and University Librarian, Otto G. Richter Library, University of Miami [9].

### Doctoral Program

Shannon embraces lifelong learning, and it shows up in her collegiate journey. To culminate all of her accomplishments in higher education as a continuous learner, Shannon began pursuing a Doctor of Education (EdD) degree with a focus on Educational Leadership at Charleston Southern University in January 2020. Covering topics such as Leadership Theory, Creativity and Innovation, Leadership and Change, and Diversity and Equity in Organizations; this program will solidify and expand on the skills she has already honed from her years of experience in leadership. She recently defended her dissertation in August 2022 and is well on her way to becoming Dr. Shannon D. Jones.

## A LEADER OF INSTITUTIONS

### Director for Libraries at MUSC

After serving in leadership roles at Virginia Commonwealth University from 2010-2014, Shannon decided to continue her leadership journey in Charleston, South Carolina, as the Assistant Director for Program Development and Resources for the Medical University of South Carolina (MUSC) Libraries. As Sandra Franklin shares in her testimonial, Shannon's recruitment to MUSC was not just because of her demonstrated ability to lead departments and divisions but also because of her potential to lead whole libraries. Shortly after arriving at MUSC, Shannon transitioned into Director of Libraries in 2015 with the full support of her predecessor Dr. Thomas G. Basler. Since 2015, Shannon has led several essential library initiatives, including a library remodel, reduction of the print collection footprint to aid in the expansion of study spaces, integration of the MUSC health system into the Libraries licenses, increasing BIPOC representation in library faculty, and staff and transitioning her library faculty and staff from onsite to remote work and back to onsite during COVID-19.

### Region 2 Director for the Network of the National Library of Medicine

Ten years after completing the NLM/AAHSL Leadership Fellows program, Shannon's leadership impact would expand beyond her directorship at MUSC. In 2021, Shannon gained an additional leadership title as the Director of the Network of the National Library of Medicine (NNLM) Region 2, also being the first African American woman in the role and the only BIPOC leader to helm a regional medical library in the 2021-2026 cohort (RML) [10]. This highly competitive federal funding opportunity for academic health sciences libraries helps to serve as a regional anchor to the National Library of Medicine. In fact, there are only seven NNLM libraries in the United States and US Territories [11].

To serve as an RML requires having the capacity, resources, and bold vision of extending the reach of the National Library of Medicine in providing training, grant resources, and support to academic and public libraries as well as community-based organizations with a focus on health literacy and promotion. In having MUSC serve as the Region 2 library, the impact and reach of Shannon's leadership, now expanded into libraries across the Southeast and Caribbean, including Alabama, Florida, Georgia, Mississippi, South Carolina, Tennessee, the Commonwealth of Puerto Rico, and the U.S. Virgin Islands [12]. In stepping into this new role, Shannon's legacy to positively influence and improve health information, access, and outcomes to historically excluded communities have come full circle.

## A LEADER IN LIBRARIANSHIP

### Committee Work and Service

Shannon has been a demonstrated leader, serving in a variety of leadership roles and memberships across libraries, on committees, advisory boards, and workgroups. At MUSC, she recently served on the Advancement, Recruitment, and Retention of Women (ARROW) Awards Committee, the Educational Resources and Infrastructure Liaison Committee on Medical Education (LCME), the Imagine MUSC 2020 Strategic Planning Team, and dozens of university, college-level, and MUSC Libraries search and screening committees for the hiring of diverse faculty and staff.

Shannon joined the American Library Association (ALA) in 2001, starting as a Spectrum Scholar and serving on many committees that gave her opportunities to showcase her varied interests and leadership skills. Committees she has been involved with include the Association of Colleges and Research Libraries (ACRL) Health Sciences Librarians Interest Group, the Black Caucus of the American Library Association (BCALA), and the Office for Diversity, Literacy and Outreach Services (ODLOS); all of which she has served and lead in various capacities. She is currently most active with the Reference and User Services Association (RUSA), where she is currently a member of the Budget and Finance and Nominating Committees; as well as the Emerging Technologies Section of RUSA, where she served as Vice Chair/Chair-Elect, Chair and Past Chair from 2019-2022.

She continues to serve on a number of advisory boards, including the Library Advisory Board of the New England Journal of Medicine (NEJM). She is also currently on advisory boards for several Institute of Museum and Library Services (IMLS) funded projects and served as a peer reviewer for the IMLS-funded Coronavirus Aid, Relief, and Economic Security (CARES) Act Grant in 2020 and the Laura Bush 21st Century Librarian Grant in 2021.

Shannon has been an active member of several other local, regional and national organizations, serving on numerous committees. As a member of the Association of Academic Health Sciences Libraries (AAHSL), she served most recently as a member of the Diversity and Inclusion Committee and on the Program and Education Committee as Chair from 2017-2020 and is currently on the Education Program Planning Team in 2021. At the National Library of Medicine, she has contributed to their Friends of the National Library of Medicine Program Committee as the 2021-2022 Co-chair of the Library Symposium Planning Subcommittee and as a reviewer for several Special Emphasis Panel/Scientific Review Groups in collaboration with the National Institutes of Health (NIH). She is currently a member of the Mid-Atlantic Chapter (MAC) of the Medical Library Association's Diversity and Inclusion Committee, the Southern Association of Colleges & Schools Commission on Colleges (SACSCOC) Substantive Change Committee, and a member of the Partnership Among South Carolina Academic Libraries (PASCAL) Board of Directors since 2018, serving as Chair-Elect, Chair, and Past Chair from 2019-2022.

### Library Journal Mover & Shaker

In 2021, the Library Journal gave Shannon the coveted title of Mover & Shaker/Change Agent [13]. The interview title succinctly describes Shannon's overall mission with everyone she's had an opportunity to meet: “[l]ift as you climb” [14]. Shannon's passion for lifting up those that come after is evident in the testimonials in this writing and anytime her name is spoken in medical librarianship circles. She has sought out individuals who otherwise would not have had a fair opportunity to enter librarianship, medical librarianship, and even leadership opportunities. She has shown mentees how to become mentors and raise up a village of future powerhouse librarians. She has also lifted up important topics on bias, privilege, and inequities faced by historically excluded and marginalized identities. She has also rallied others to the cause of advocacy and activism.

## CONTRIBUTIONS TO THE MEDICAL LIBRARY ASSOCIATION

### New Members Caucus

Shannon's first significant contribution to the Association was helping to create the New Members Special Interest Group (SIG) in 2005. The original purpose of the New Members Special Interest Group was to provide a forum for librarians with less than three years of experience to engage in information exchange and discuss information important to new librarians and new MLA members. The goal of the New Members SIG was to foster learning, cooperation, and support amongst new librarians and new MLA members; however, membership was open to any librarian interested in issues and concerns affecting new librarians and new members. As part of supporting new members, she and inaugural co-convener Tomeka Oubichon established a New Members Column in MLA News that shared relevant information to help new members navigate the Association [15]. At the time of its founding, the New Members SIG was sponsored by the Leadership and Management (LMS) section before transitioning to a caucus in 2019.

### African American Medical Librarians Alliance (AAMLA) Caucus Chair & Mentor

Since joining in 2003, Shannon has made a resounding impact within the membership of the Medical Library Association. Where she has truly made, the most influential change has been in her home caucus, the African American Medical Librarians Alliance (AAMLA). AAMLA has served a critical role in creating a community with library workers of African American and Black descent since its founding as an informal group during the late 1980s. For those not familiar with this caucus, the purpose of AAMLA is:

**“…providing leadership and support on issues such as recruiting and retaining diverse librarians, developing mentoring and leadership for minority librarians, and creating a repository of information on the expert skills and special abilities of minority librarians in Medical Library Association (MLA), particularly those of African American descent.**

**AAMLA stands committed to bringing the challenges and issues of minority librarians and information professionals to the forefront. Our role is to help all medical librarians and information professionals understand and appreciate the dynamics of cultural diversity, as well as to recognize and address the needs for cultural competence in the healthcare environments.” [16]**

As a member of the caucus for well over two decades, Shannon's goal with AAMLA was and continues to be to ensure that the contributions of the Black librarians who had come before her were acknowledged, valued, and amplified as well as showcasing how Black librarians were actively contributing to work in health sciences libraries. For Shannon, it has been all about legacy building. In order to see these visions become a reality, Shannon has taken on important roles within the caucus. She has served as caucus chair and helped to manage and jump-start several caucus initiatives, including the Virtual Engagement Committee, MLA Reads, Virtual Chat & Chew, the AAMLA website and Twitter accounts, and spearheading the creation of a timeline of Black librarian contributions to MLA.

The Virtual Engagement Committee (VEC) was created for three purposes. Firstly, VEC created a service opportunity for Black librarians. Secondly, VEC created value-added educational content for Black librarians who were unable to attend the annual MLA Conference. Thirdly, the VEC was an opportunity to provide a national platform for Black librarians to share/present their work and wisdom in libraries. In her capacity, Shannon facilitated programming ideas, speaker lineups, designing graphics, and setting up a registration system.

The Virtual Chat & Chew was launched in March 2020 as a virtual community that came together in response to the COVID-19 pandemic. The first meeting was held to discuss how to adjust to remote work and share advice to help alleviate the stresses of transitioning to a new normal. Early meeting topics included tech tools and web conferencing platforms that became indispensable during remote work, warnings on how to remain vigilant of racially based Zoom bombings, and critical conversations on how COVID-19 adversely impacted Black communities. The Virtual Chat & Chew became a much-needed safe space for important discussions that seldom took place in predominantly white environments. However, the difficult subject matter also came with opportunities to share accomplishments and achievements among members, establish an informal information exchange on COVID-19-related institutional policies, participate in gamification activities, hold book discussions, host special guests, and even a holiday gift exchange [17]. The Virtual Chat & Chew continues to be a welcome space for Black & African American librarians across the United States.

Shannon also served as AAMLA's Web Coordinator starting in 2004 and was instrumental in moving the site to MLAnet from the University of Buffalo, where Ophelia Morey hosted it. She was responsible for keeping the website up to date and later co-designed with author Kelsa Bartley, its spinoff using Google Sites which allowed for a more robust platform to showcase the unique perspectives and voices of Black librarians in MLA [18]. The site lists information about AAMLA, position statements, honors and awards, upcoming events, and links to recordings of past events.

### MLA Reads

One of the instrumental ways she has rallied others to the cause of making diversity, equity, and inclusion at the forefront of medical librarianship has been through the initiative she co-founded, known as MLA Reads. A virtual book club initiative, MLA Reads was founded by Shannon Jones and fellow creator Kelsa Bartley after conference participants at the 2018 Medical Librarian Association Conference shared their need for a safe space to better understand their own relationship with bias, privilege and explore other topics on diversity, equity, and inclusion [19]. The birth of MLA Reads could not have come at a more urgent time in our history, during which the COVID-19 pandemic and reckonings of racial injustices magnified the health disparities that have long existed in the United States. The success of MLA Reads has led to annual gatherings where participants have been able to read a new selection of books each year, discuss them in groups and apply the lessons learned in their personal and work life. In Shannon's own words, MLA Reads both help to “[acknowledge diverse lived experiences” and “[a]dopt a growth mindset” that helps to lead readers to action in addressing the visible and hidden barriers that come with bias and discrimination in libraries [20]. Not only did Shannon help facilitate this important reading initiative, but she also wrote and co-edited, a book that further addressed issues related to diversity in libraries, titled, *Diversity and Inclusion in Libraries: A Call to Action and Strategies for Success*, with mentor Beverly Murphy in 2019 [21]. The MLA Reads program is also discussed in greater detail in the book chapter “Braving our Blind Spots: Using a Virtual Book Discussion Group to Continue Conversations on Implicit Bias in Libraries” in the book *Implementing Excellence in Diversity, Equity, and Inclusion: A Handbook for Academic Libraries* [22] that she co-authored with Kelsa Bartley and four other members of the MLA Reads Planning Team.

### MLA President

One would think that Shannon's mission to lead and influence the profession would stop at the Region 2 title, but it's unlike Shannon to slow things down. That same year, in 2021, Shannon became President-Elect for the Medical Library Association, serving as only the second African American to be in the role. The first was her mentor, Beverly Murphy, in 2019 [23]. Now in this capacity, Shannon serves on both the Board of Directors and the Executive Committee overseeing the goals and initiatives of the Association [24].

In her capacity as President, Shannon has continued her mission to improve diversity and wellbeing among the membership and emphasized this through programming such as “Be Well MLA,” her first initiative, a monthly series of member-created webinars sharing strategies for improving one's own health and wellness. Shannon wants to continue striving toward and championing wellness and well-being. She wants us all to do a better job of taking care of ourselves, and she understands that slight changes to our care make a difference in our overall well-being. Secondly, Shannon wants to ensure that MLA continues to progress in the areas of Diversity, Equity, Inclusion, and Belonging. This has been important to her since she first joined MLA in 2002. The Association is not where we were twenty years ago, but we have much more work to do to make sure all MLA members feel like their unique perspectives and voices are heard. Shannon stated in her Presidential Inaugural address, “We are not where we need to be, but we are actively working on getting it right for ALL of our members. We are doing the work to walk the walk, and I am proud of us [25].” Thirdly, Shannon wants to build a better future by including the narratives of all health sciences/medical librarians. She also stated, “I look forward to contributing to the work MLA members do to capture the voices, perspectives, contributions, and history of members traditionally left on the margins of the Association's life [26].” Shannon is looking forward to celebrating MLA's 125 anniversary in Detroit and is most excited about MLA's commitment to making sure all MLA members' voices are heard in the historical narrative. She is looking forward to contributing to MLA members' efforts to capture the voices, perspectives, contributions, and history of members who the Association historically excluded. Shannon's fourth initiative is encouraging members to embrace the concept of radical empathy and for us to consider radical empathy to be the new normal. After Shannon read the book, *Radical Empathy: Finding a Path to Bridging* by Terri Givens, it solidified the importance of the six tenets of radical empathy that Givens outlined in the book. Givens defines radical empathy as moving beyond an understanding of others' lives and pain to understand the origins of our biases. In practice, the tenets of radical empathy embraces a willingness to be vulnerable, opening yourself to the experiences of others, taking action, becoming grounded in who you are, practicing empathy, creating change and building trust [27].

Shannon's rally cry for all MLA members is to do as the pop icon Beyonce suggested and get “In Formation” if we want to accelerate this work so that we as a membership body are walking in lockstep, hand in hand, to confront issues facing our libraries.

### MLA Fellow

In the summer of 2022, Shannon's achievements as an academic health sciences librarian and leader would culminate in being accepted as a Fellow of the Medical Library Association (FMLA), essentially the Hall of Fame for accomplished and impactful medical librarians. Shannon's impressive career and accomplishments now sit along with other prominent library figures like Kristine Alpi, Rachel K. Anderson, Estelle Brodman, Naomi C. Broering, Ana Cleveland, Janet Doe, Sandra G. Franklin, Teresa L. Knott, Beverly Murphy, MJ Tooey, and others who've made a significant impact on the field [28].

### The Firsts: A Tale of Two African American MLA Presidents

When Beverly Murphy became the first African American president, it was Shannon who wrote her presidential biography [29]. Here we take the opportunity to share in Beverly's own words her relationship and mentorship of Shannon over the course of more than two decades:

**“I've known Shannon for 22 years, and for all those years, she has been active in librarianship, formulating ‘sustained and outstanding’ contributions to the field and to MLA as a visionary, leader, and collaborator.**

**Shannon and I met at an Association of North Carolina Health and Sciences Libraries (ANCHASL) meeting in 2000 while she was still in library school and working at NC State. From that moment, she was someone that I knew I would have a lifelong friendship with, both professionally and personally. She filled the room that day, and I wanted to know more about her. So, I offered to mentor her, knowing it would be a mutually engaging experience for both of us. Since then, we have shared a strong tradition of promoting excellence in health sciences librarianship.**

**When Shannon came onto the Medical Library Association (MLA) Board in 2018, I made the decision, as MLA President, to appoint her as MLA Treasurer. I can honestly say she was the right person to fill that leadership role, as the financial transition for the MLA Communities (especially the former Sections) was extremely challenging. But she handled it like a champ - remaining calm, explaining and re-explaining the process, and establishing consensus to ensure members' needs would be met. She extended that role by chairing the MLA Finance Committee from 2019-2021. I was extremely proud to learn in 2021 that my mentee would be following right after me as the second African American President of MLA, a distinction that has a special place in my heart for many reasons. In our ongoing peer mentorship, I have looked forward to being by her side and witnessing firsthand her achievements while in this role.**

**I've witnessed Shannon be a fierce advocate within the profession for people from marginalized communities and through her service on the MLA Scholarship for Minority Students Jury, the Black Caucus of the American Library Association, as an ALA Spectrum Scholar, and as a member of AAHSL's Diversity and Inclusion Committee, as well as MLA's Professional Recruitment and Retention Committee and the African American Medical Librarians Alliance (AAMLA) Caucus.**

**Working with Shannon on co-editing *Diversity and Inclusion in Libraries: A Call to Action and Strategies for Success* was a unique and awesome experience. Since it was published in August 2019, we have sold over 850+ copies and the book is in 250 libraries around the world. It has been listed as one of Our Favorite Books About Libraries and Librarians by ilovelibraries, an initiative of the American Library Association to promote the value of libraries and librarians. This has shown me just how impactful a book written by people from the BIPOC community can be. It would not have been the same experience had I not partnered with Shannon. We enjoyed our collaboration on the first book so much that we are now working on co-editing a second book on *Cultural Humility in Libraries*, set for publication in 2023.**

**We've had many other collaborations including an interview in January 2021 conducted by Ray Pun, Special Libraries Association Leadership and Management Development Community member, where we shared our experiences and thoughts on Diversity and Inclusion in Libraries; Press, Play, Connect MLA podcast in September 2021 on “Diversity, Equity, and Inclusion in Libraries;” and a LibVoices podcast on “Representation, Retention, and Trailblazing” in October 2021 [30][31][32]. Our relationship has not only been one of growth and engagement, but one of mutual respect for the work that we do on behalf of diversity, equity, and inclusion.**

**Shannon's notable leadership is demonstrated by the plethora of elected and appointed offices, committees, task forces, board memberships, etc. that she has participated in, on the national and regional level. Her resume speaks for itself so I will not reiterate that here as there are way too many activities to name. I have opted instead to tell you about the essence of Shannon, the person that I know and have the utmost respect for. The person that I've watched grow over time and that continues to grow is a marvel, because her heart is open to the diversity of her colleagues and her mind is open to our ever-changing profession.**

**Among the many qualities that Shannon possesses, perhaps the most significant for our profession has been her desire to provide a forum for librarians at all levels to grow. Her empathy for people and their situations, in addition to her tenacity, courage, and compassion, has given her the ability to understand the needs of people and reach them where they are.**

**Shannon is an awesome leader, innovator, and motivator and has worked tirelessly as a volunteer for our profession and on behalf of our colleagues.”**

## TESTIMONIALS

Aside from listing out Shannon's tremendous series of accomplishments and achievements, the authors felt that it was essential to bring in additional voices that were impacted by Shannon's mentorship, leadership, and friendship. We reached out to several mentors, mentees, and frolleagues and asked them to share stories or inspiring moments. We also requested to keep it to 150 words, with nearly unanimous feedback that such a limited word count was not enough to share how impactful Shannon has been in their personal and professional lives. Below are their responses.

### Mentors

**“When Tom Basler, Ph.D., was recruiting an Associate Director, he personally told me that he was looking for his successor. I was so pleased when Shannon Jones was hired. Tom mentored her to succeed in the Medical University of South Carolina environment and to become the outstanding AAHSL director she is today. For several annual meetings, Shannon chaired the AAHSL Education Committee, and the committee offered innovative topics and renowned speakers. She is a well-respected mentor to many librarians of all races and genders and demonstrates, by example, the importance of continuous higher education. Shannon rendered extraordinary leadership to numerous organizations on her path to becoming President of the Medical Library Association. During an MLA 2022 Immersion session on Mentoring, Shannon named me as one of her role models. It pleased me to know that I had an impact on one so prominent. I am very proud of Shannon and her accomplishments.”**

- Sandra Franklin, MLS, AHIP, FMLA, Director, Woodruff Health Sciences Library, Emory University

**“Shannon is beyond a force of nature. I am not sure how she does everything – her family, Girl Scouts, her beloved pups, leading, volunteering, and just getting things done. Seeing her pictures on Facebook exhausts me! We laugh together. On occasion, she has made me snort! How undignified for directors! She takes chances on people, valuing those others might not see as valuable. She leads and teaches by example. She gathers people together to learn share, and grow. Shannon does everything with great warmth and conviction without dithering. Stepping back and reviewing her accomplishments, all you can say is “wow.” She advances with purpose, conviction, and clarity. We agree on so many things sometimes I look at her and say, “What, you too?” It is said that we chose to associate and become friends with people who are like us and who help us to be our best selves. What a frolleague!”**

- MJ Tooey, MLS, AHIP, FMLA, Associate Vice Provost Dean, Health Sciences and Human Services Library, Executive Director, Health Sciences and Human Services Library, Director, Network of the National Library of Medicine, Region 1, University of Maryland

### Mentees

**“I was matched with Shannon as a library student through the ALA mentoring program. She has been so instrumental throughout my career and has supported and mentored me in every job since I graduated. She has been invaluable in providing career support and advice and has always made time, despite her busy schedule. Her passion and drive for health librarianship has been truly inspirational and I am very fortunate to have had her as a mentor!”**

- Faith Steele, Outreach and Education Librarian, NNLM Region 1 University of Maryland, Baltimore

**“Shannon is my role model, mentor, sister and friend. Her coming into my life truly changed the trajectory of my career in health sciences. She motivates, inspires and brings out the best in me. Because she believed I could do great things; I try every chance I get. I endeavor to pay it forward and be the light she has been to me.”**

- Tamara M. Nelson, MLIS, EdS, AHIP, Associate Professor, User Services Coordinator/Senior Research & Learning Services Librarian, University of Tennessee Health Science Center

**“There's truly not enough space to expand on Shannon's mentorship, friendship, and legacy and understand how much impact she has made in the lives of others. As another frolleague, MJ Tooey points out, Shannon has a special ability to see the value in others and then help leverage that value for the betterment of themselves and the medical library profession. Had Shannon not found me as a hospital librarian tucked away in the web pages of LinkedIn, it's hard to tell where my path would have led me. In having the opportunity to meet Shannon as a potential hire, I learned a valuable lesson from her and the experience. I had long believed I would not be able to make the leap to academic librarianship because of many perceived hurdles. Becoming an academic medical librarian and a leader seemed like a forgone opportunity. Instead, my experience with Shannon made me realize that these dreams were (and have since become) very much attainable. It wasn't a matter of where this would happen, but when. Looking back, I have found this to be a lesson I reflect on often. Shannon's leadership shines not because she seeks out value in others, but because she reflects back to us what is already there and then lets you realize how much brilliant potential exists in ourselves. It's a leadership attribute that not many can say they possess, but it comes natural to Shannon, and we are certainly all better for it.”**

- Aidy Weeks MSLIS, AHIP, Assistant Professor, Director, School of Medicine Library, University of Nevada, Las Vegas

**“I met Shannon at my first ever ALA conference, at a Spectrum Scholars event at the beginning of my library school journey. I was so in awe at the time, meeting a Black librarian who was also a library director so early in my career was such an inspiration. Meeting her then, I had no idea how much of an impact she would have on my trajectory in librarianship, in such a short timeframe. We were reintroduced at the MLA conference and since then, she has been a great mentor, an amazing advocate, a supportive cheerleader, and a wonderful friend to me. She genuinely cares about my interests, continues to challenge me to build my strengths and expand my talents, creates opportunities for me to thrive and flourish in libraries, and advocates for me in places I probably would not be otherwise. What amazes me most about Shannon, is that she is constantly learning-about people, about libraries; not only about the things that impact her directly, but also for the benefit of those around her. She cares deeply for her “people''-whether they are at MUSC, MLA, AAMLA, librarian colleagues across libraries, Girl Scouts, family, friends, and pooches. It is her fierce commitment to excellence, and to make sure that her tribe is just as excellent along with her-in any way she can make help make that possible; that makes her a strong and compassionate leader, in libraries and in life.”**

- Kelsa Bartley, MSI, AHIP, Assistant Professor, Education, and Outreach Librarian, University of Miami Miller School of Medicine

### From MUSC and beyond

**“When people ask me about Shannon, there is only one word I use to describe her: fair. I've never worked with someone who put so much effort into trying to make sure everyone was treated equally and given the same opportunities regardless of their job title. She does not show favoritism, nor does she let anyone fall behind. She also goes out of her way (although I don't think she sees it as doing so) to help mentor newer librarians and give them career advice on how to set themselves up to be successful. I am positive a good number of the people who are reading this article have learned something from her at some point over the years. I do not take it for granted that I get to work alongside her every day. She's my “sunshine” and I am incredibly fortunate to be in the position I am.”**

- Heather N. Holmes, MLIS, AHIP Associate Director of Libraries, Medical University of South Carolina

**“Many people may not know this, but the title of “The Shannon Jones,” was started by me! Several folks like myself came to work for MUSC because of Shannon's stellar reputation and achievements, particularly as a Black woman leader in the information profession. Shannon worked tirelessly with a team at MUSC to apply and then be funded as the Region 2 Regional Medical Library in Charleston. Together, they won a prestigious government-funded cooperative agreement to spread trusted health information and resources to members throughout the southeastern region. My team continues to benefit greatly from the opportunity to work for the RML at MUSC.”**

- Lorin Jackson, Executive Director for the Region 2 Regional Medical Library, Medical University of South Carolina (MUSC)

**“In 2019, I met Shannon at my first BCALA membership meeting reception during ALA midwinter. In that same year, I started to get to know Shannon better as a committee member of AAMLA's virtual engagement committee. When I began to watch her leadership abilities, organizational skills, and tech-savvy talent, it excited and inspired me. At the beginning of 2020, when the COVID-19 pandemic began, Shannon took her care of her AAMLA colleagues to another level by hosting weekly “chat and chews.” These meetings were so helpful to me. I will end with this—I admire how Shannon uplifts others by seeing their potential and how she brings them alongside her.”**

- Jamia Williams, MLS, Health Sciences Librarian, Drake Memorial Library, SUNY Brockport

### Other Directors

**“Among Shannon Jones' greatest strengths is an ability to identify and embrace emerging needs and trends whether in health sciences librarianship, leadership, or technology. The first time I observed Shannon presenting at a conference, she facilitated a panel on millennials in the workplace in 2004. She was attempting to bridge understanding between generations and smooth integration of millennials into the profession. Since then, she has continued working toward more inclusive professional organizations. Throughout her career, she has tried to engage a wide range of people and to foster the development of understanding, empathy, and respect for individual differences. There is so much that I admire about Shannon. Besides being highly intelligent, resourceful, and innovative, Shannon is warm and personable. She is a truth teller. If you want an honest opinion, you will always receive one from Shannon. She mentors as readily as she breathes. A strong proponent of setting professional and personal goals, Shannon has a record of achievement that is outstanding. What is sometimes less obvious are the number of colleagues that Shannon has helped achieve their goals and advance their careers through her support and counsel.”**

- Teresa L. Knott, MLS, MPA, AHIP, FMLA, Associate Dean, VCU Libraries, and Director, VCU Health Sciences Library, Virginia Commonwealth University

**“Shannon Jones is one of those people who demands immediate respect by virtue of her presence and her thoughtful commentary. You cannot help but think, ‘She knows what she's talking about’ when in conversation with her. This is true if the conversation is friendly and immediate or at the national or discipline level. Obviously, to anyone that knows her, what I'm describing is ‘gravitas,’ and she possesses this skill, the ability to take over a room, with humor and confidence. I have always admired her for this. And her collection of glasses.”**

- J. Dale Prince, MA, MLS, AHIP, Director of Libraries, LSU Health Sciences Center - New Orleans

**“Shannon inspired me when she spoke during a New Library Directors event about issues she was having with staffing at her library. She spoke in a way that did not disparage any of her staff but also authentically described problems. It was an example to me of how it is possible to speak about problems or concerns in a manner that is honest but does not put others down. Related to this, Shannon spoke at a MLA programming event one of my coworkers attended. My co-worker shared with me how she was impacted by Shannon's comments. My co-worker said they had a better understanding of the issue after hearing Shannon speak, and they were impressed with Shannon's authenticity. I strive to be authentic in my words and actions, and Shannon is one of the role models I look to in this area. There are definitely times when I ask myself ‘What would Shannon do?’ when I'm working through a tough situation.”**

- Melissa De Santis, MLIS, AHIP, Director, Univ of Colorado Anschutz Medical Campus

**“When Shannon is leading, I know it's going to be fun! And, joyful, thoughtful of all involved, intentional in practice, and always in search of ways to grow the circle wider. I met Shannon at Transforming Libraries Using Implicit Bias Training, an MLANET 2018 session she presented with Kelsa Bartley. They shared copies of Blind Spot: Hidden Biases of Good People, and I was so excited about their idea for virtual book discussions open to all – now the annual MLA Reads. As a library and NNLM region director; through programs, publications; as Treasurer and our 2nd African American President of MLA, Shannon has made a transformative impact throughout our profession, leading a movement that tangibly increases equity, inclusion, and connections across race, gender, sexual orientation, people with disabilities, nationalities, more. Shannon brings us all along and inspires us to keep equity and inclusion at the forefront in all we do. I'm so grateful for her leadership.”**

- Ginny Pannabecker, MA, MA (IRLS), Assistant Dean and Director, Research Collaboration and Engagement, University Libraries, Virginia Tech

### Frolleauges

**“I met Shannon many many moons ago. I have had the opportunity to work with Shannon on some projects with BCALA, and MLA and she taught me a lot about Health Sciences when I was working as a Health Sciences Librarian. She has been a great mentor and friend.”**

- Jahala Simuel, MLS, MIS, High School Librarian with District of Columbia Public Schools

**“In all honesty, Shannon has been an inspiration to me since the day I first met her. First, let me start with the beautiful and amazing person she is. Shannon is one of the most compassionate, authentic, confident, fun, intelligent, graceful, fashionable, and giving people I have ever met. She is a strategically focused, detailed, transformational and Inclusive leader, as well as a reliable friend. I am inspired by how she takes the time to check-in, encourage, and motivate me to keep pressing on. She is always transparent and honest with me, and I sincerely appreciate her words of wisdom and unwavering support throughout my career and for her helping me to advance in MLA and secure my current position as Associate Director. I am in awe of how she has the energy and time to lead her library, lead and be active in professional organizations, work on her doctorate, lead a Girl Scout troop, be a mom to Cooper and Ram, travel and spend time with family and friends, and still be there whenever any of us need her. She is sincere, influential, strong, positive and a joy to be around. She perseveres through the many challenges she faces and never lets anything get her down. Shannon is the glue that keeps AAMLA strong and her sister colleagues on the right track to achieving great heights and soaring as leaders. I would not be where I am professionally if God had not let our paths cross years ago. I treasure her friendship and investment in me over the years. I pray I will evolve into an amazing leader like Shannon. She has been a wonderful example of leading with courage and empathy. MLA will be a more inclusive, engaging, and welcoming space as Shannon leads us forward. She values each of us and is committed to her role as President.”**

- Tara Douglas-Williams, MSLS, AHIP, Associate Director, Emory University

**“Shannon has always taken time in her busy schedule to mentor me. I met her during the 2018 MLA conference at a wonderful immersion session on Implicit Bias. She was so joyful and excited to be sharing the work. Later I teamed up with her for the MLA Reads project and have become a member of the core planning team. Shannon is full of life, positive energy, and ideas! She has guided me in ways that I am forever grateful for, and I appreciate all the time and silly questions she has had to endure with me. She has included me in publishing projects and committee work that have really expanded my network. I consider her a great mentor/friend, and I'm glad she is our MLA President!”**

- Dede Rios, MS, Ph.D., AHIP, Director of Optometric & Clinical Library Services, University of the Incarnate Word

## CONCLUSION

Though we have highlighted Shannon's journey into librarianship and medical librarianship and her accolades as a library leader and change agent, it's really through her own words that Shannon's influence within the profession shines brightest. In her interview, “Preparing the Next Generation of Leaders: From NLM Associate Fellow to Health Sciences Library Director,” Shannon remarks on what we conclude to be her hallmarks as a leader and compassionate human being [33]:

**“Dream BIG: Lead my staff towards achieving our big, bold vision of making our library the place on campus where people come to do their best work.**

**BE the CHANGE: Serve as a positive role model for librarians who are following me in the profession, especially those from traditionally under-represented groups.**

**Love THY Self: Practice self-care by following the instructions that flight attendants give passengers before takeoff: Putting on my oxygen mask before helping others.”**

**Shannon's formula for success is to dream big, be the change, and love oneself. On the surface appears only to have catapulted her to positions and opportunities not often provided to BIPOC women. In reality, however, Shannon's perseverance, empathetic leadership, and desire to uplift others are possible because of the conviction behind this framework. There is no story within the medical librarianship field that includes Shannon and omits her heart's work to lift up the profession for everyone.**
